# Objectively Measured Daily Physical Activity and Postural Changes as Related to Positive and Negative Affect Using Ambulatory Monitoring Assessments

**DOI:** 10.1097/PSY.0000000000000485

**Published:** 2017-06-20

**Authors:** Daniel Aggio, Karen Wallace, Nicola Boreham, Aparna Shankar, Andrew Steptoe, Mark Hamer

**Affiliations:** From the UCL Department of Primary Care and Population Health (Aggio), UCL Medical School; Department of Epidemiology and Public Health (Wallace, Boreham, Steptoe, Hamer), University College London; Population Health Research Institute (Shankar), St. George's, University of London; and School of Sport, Exercise and Health Sciences (Hamer), Loughborough University, Loughborough, United Kingdom.

**Keywords:** mood, negative affect, physical activity, positive affect, sedentary behavior, **BMI** = body mass index, **CI** = confidence interval

## Abstract

**Objective:**

The aim of the study was to determine whether objectively measured daily physical activity and posture of sitting, standing, and sit-to-stand transitions are associated with daily assessments of affect.

**Methods:**

Participants (*N* = 51, 49% female) wore ActivPal accelerometers for 24 h/d for seven consecutive days. Time spent sitting, standing, and being physically active and sit-to-stand transitions were derived for each day. Participants also completed a mood inventory each evening. Multilevel models examined within- and between-person associations of daily physical activity with positive and negative affect, adjusting for age, sex, body mass index, education, and sleep duration.

**Results:**

Within-person associations showed that a 1-hour increase in daily physical activity was associated with a decrease in negative affect over the same day (*B* = −0.11, 95% confidence interval [CI], −0.21 to −0.01). Between-person associations indicated a borderline significant association between higher average daily physical activity levels and higher positive affect (*B* = 1.85, 95% CI = −0.25 to 3.94). There were no between- or within-person associations between sitting, standing, and sit-to-stand transitions with affect.

**Conclusions:**

Promoting physical activity may be a potential intervention strategy to acutely suppress negative affective states.

## Abstract 2

Supplemental digital content is available in the text.

## INTRODUCTION

Physical activity has been associated with numerous psychological health benefits, such as a reduced risk of depression and anxiety in adults (1–4). Most studies use single measures of physical activity and depression taken at one or two time points. However, we know that individual affective states vary on a daily basis (5), and therefore, measures taken at a single time points do not account for within-person variation. Recent evidence has been accumulating on the effects of acute bouts of physical activity on affective states within individuals (6–9). A meta-analysis of 158 published and unpublished studies showed that acute bouts of structured physical activity are associated with increased positive affect (9). Recent findings in free-living naturalistic settings have confirmed these findings (7,8). Although there is consistent evidence that acute bouts of physical activity increase positive affect, evidence on the acute effects on negative affect remains inconclusive (7). One possible explanation for these inconsistencies may be the different techniques used to measure physical activity. A recent systematic review including 14 studies examining these associations identified only six studies using objective measures (7). Self-report measures are prone to measurement error because of recall bias and may reveal different associations with affective states when compared with objective measures. A recent study from the Health Survey for England found similar positive associations for objectively and subjectively measured sedentary time with psychological distress, but only self-reported, and not objectively measured, moderate to vigorous activity was associated with lower risk of psychological distress (10). The use of self-reported measures of physical activity and sedentary behavior may provide useful insights on specific contexts of behavior that are more strongly associated with affective states but compared with objective measures may be less accurate for quantifying total time spent active or sedentary.

Strengths of more recent studies include the use of objectively measured physical activity, which provide a more accurate estimate of activity levels (11–16). However, these studies typically use accelerometers that are unable to differentiate between sitting and standing/light ambulatory movement and are therefore less accurate for categorizing sedentary time, standing, and breaks in sedentary time (17,18). In contrast to physical activity, sedentary behavior is thought to be detrimental to psychological well-being (10,19,20), independent of physical activity levels. Despite this, few studies have quantified how sedentary behavior is associated with acute affective states. Furthermore, recent evidence has emerged demonstrating that standing may be associated with improvements to certain quality of life measures including reduced fatigue (21) and increased social functioning (22). Through a cognitive appraisal process, even activities at a lower intensity can be beneficial for affective states (23). However, the acute and habitual effects of standing and sit-to-stand transitions on affective states have not been explored. Methods that can detect postural allocation may be more reliable for determining sitting and standing time, which may help us better understand the associations of sedentary time and standing/light activity with affective states (17). To the best of our knowledge, there have been no studies investigating the within-person associations between activity levels and daily assessments of affective states with appropriate objective measures of sedentary time. We hypothesized that higher overall and daily increases in free-living physical activity, standing, and sit-to-stand transitions measured across several days would be associated with more favorable affective states. Conversely, higher overall sitting time and daily increases in sitting time would be associated with less favorable affective states.

The primary aim of the present study was to determine whether objectively measured free-living daily physical activity, sitting time, standing, and sit-to-stand transitions were associated with daily assessments of affect. This was investigated on a between-person basis, assessing the relationships between average physical activity across several days and aggregated daily positive and negative affect, and in within-person analyses exploring associations between fluctuations in objective physical activity and affective states across days.

## METHODS

### Participants

Participants (*N* = 51, aged 19–41 years) were recruited as part of a randomized controlled trial that examined the effects of consuming a tea component (theanine) on changes in blood pressure during acute mental stress. All data analyzed in this study were collected before the intervention phase of the trial. Participants were recruited from University College London, and 70% were students. Enrolment in the study required low caffeine consumption, no history of mental illness, nonsmoking, a body mass index (BMI) of less than 30, and a General Health Questionnaire 12 score of less than 4. Participants provided written informed consent to participate in the study, and ethical approval was obtained from the University College London Research Ethics Committee. All data were collected between March and July 2015.

### Physical Activity

Participants were fitted with an ActivPal accelerometer attached to the middle of the thigh, which was worn all day for 7 consecutive days, including during sleep. Devices were fitted with a waterproof dressing allowing them to be worn during bathing, swimming, and other water-based activities. The ActivPal is a validated uniaxial accelerometer, which produces a signal related to the angle of inclination of the leg to estimate time in different postures (e.g., sitting, standing, stepping) and number of sit-to-stand transitions, based on proprietary algorithms (24). The ActivPal demonstrates good agreement with video observations of sitting, standing, and walking (mean percentage differences in time spent in postural allocations ranged from −2.0% to 1.4%), and also excellent interdevice reliability (intraclass correlation coefficients ranged from 0.79 to 0.99) (24). Activpal data are recorded at a frequency of 20 Hz.

After completion of the 7-day wear, ActivPal data were downloaded and processed using the ActivPal interface program and exported into Excel. Time-stamped data were summarized in 15-second intervals. Days with unusual episodes were removed from analyses based on visual inspection of the raw data. Days where participants reported removing the device for periods longer than 60 minutes were also removed from analyses, an approach similar to previously published procedures (25). The first day of accelerometry data was also excluded because of the potential reactivity effect of wearing the device. Time spent sitting, standing (while not stepping), stepping (referred to as physical activity herein), and frequency of sit/stand transitions were derived for each participant for each day during self-reported waking hours. Daily means were also calculated for all activity variables. Participants with no valid days of accelerometer data were excluded from analyses.

### Affective States

Positive and negative affect were assessed using a modified version of the positive and negative emotional style scales devised by Cohen and colleagues (26). The measures were administered online using a university online survey system and were completed each evening for the 7 days of accelerometry.

Participants were asked to rate the degree to which they felt a number of emotions that day on a five-point Likert scale, ranging from 0 (you have not felt this at all today) to 4 (you felt this way a lot today). Adjectives were derived from a factor analysis of affect items (27). Daily positive affect scores were derived by summing responses to positive emotions comprising subscales of well-being (happy, cheerful), vigor (lively, full of pep), and calm (calm, at ease). Daily negative affect scores were also generated by aggregating subscales related to anger (angry, hostile), depression (sad, unhappy), and anxiety (tense, on edge). There is evidence that some physical feeling states may be distinct from overall negative and positive affective states (13). Thus, in a supplementary analyses, we distinguished between activated and deactivated positive and negative affect; scores for the vigor and calm subscales were used to describe activated and deactivated positive affect, respectively, whereas the anxiety and depression subscales described activated and deactivated negative affect.

### Covariates

Covariates were selected a priori on the basis of existing evidence in the field. During screening, participants reported personal characteristics including age, sex, and highest educational attainment (A levels, undergraduate degree, postgraduate degree). Height was measured to the nearest 0.1 cm using a Leicester Height Measure with participants in the Frankfort plane. Weight was measured in light clothing to the nearest 0.1 kg using Tanita electronic scales. BMI was calculated as weight (kilogram) divided by height (meter) squared. Participants also reported daily bedtime and wake times, from which sleep duration was estimated.

### Statistical Analysis

Multilevel models were used to examine the association between objective activity variables and positive and negative affect. Multilevel models are commonly used for analyzing repeated measures as they account for clustering of repeated measures within the same individual (28). We initially ran random intercept only models for positive and negative affect without predictor variables, revealing intraclass correlation coefficients of 0.72 and 0.70, suggesting that 28% and 30% of the variation in positive and negative affect lies within people, respectively. For activity variables, intraclass correlation coefficients ranged from 0.21 to 0.44, suggesting that most of the variation in activity lies within people. To examine whether differences in overall levels of activity (between-person) are associated with affective states, mean time across the week (hour) was calculated for each activity. To establish whether day-to-day changes in activity levels (within-person) were associated with affect, mean-centered values (daily value − weekly mean) for each of these variables were created (29). To explore the within- and between-person effects, subject-specific means (averaged across the week) and mean-centered values were included as covariates in the models (29,30). This allowed us to address in the same model (*a*) whether participants with more/less daily physical activity on average experience more/less positive or negative affect and (*b*) whether fluctuations in daily physical activity are associated with individual-level changes in affect on the same day. Negative affect scores were positively skewed and were square root transformed before analyses. Skewness and kurtosis of affect measures were 0.3 or less and less than 3.0, respectively. Separate analyses were conducted for positive and negative affect as outcomes. First, unadjusted models were run (model 1) as well as models adjusting for age, sex, BMI, sleep duration, and highest educational qualification (model 2). We did not mutually adjust for physical activity when fitting models with sitting time as the main exposure and visa verse to avoid collinearity. All analyses were conducted using STATA Version 12 (Stata Corp, College Station, Tex).

## RESULTS

A total of 51 participants were included in the analyses (male, 51.0%; M [SD] age = 24.0 [4.7] years), because six participants of the original sample did not wear the accelerometer at all. Most of the analytic sample (80%) provided at least 5 valid days of accelerometry data and 90% reported their positive and negative affect on all 7 days. Compared with participants with less than 6 days of valid accelerometry data, participants with complete accelerometry data spent significantly more time physically active (1.9 versus 0.9 h/d, *p* < .001), standing (3.8 versus 2.4, *p* < .001), and less time sitting (10.2 versus 12.6, *p* < .001). Sample characteristics for participants with at least 1 day of valid accelerometry are shown in Table 1. It can be seen that participants were typically in their 20s, were highly educated, and had normal body weights. Participants spent an average 10.4 h/d sitting, 3.8 hours standing without moving around, and 1.8 hours in physical activity. The number of transitions between sitting and standing varied widely but averaged 46.1 per day. We observed moderate to strong correlations (*r* = .40–.69) between activity variables (Table S1, Supplemental Digital Content 1, http://links.lww.com/PSYMED/A399) and a moderate negative correlation between measures of positive and square-root transformed negative affect (*r* = −.54).

**TABLE 1:**
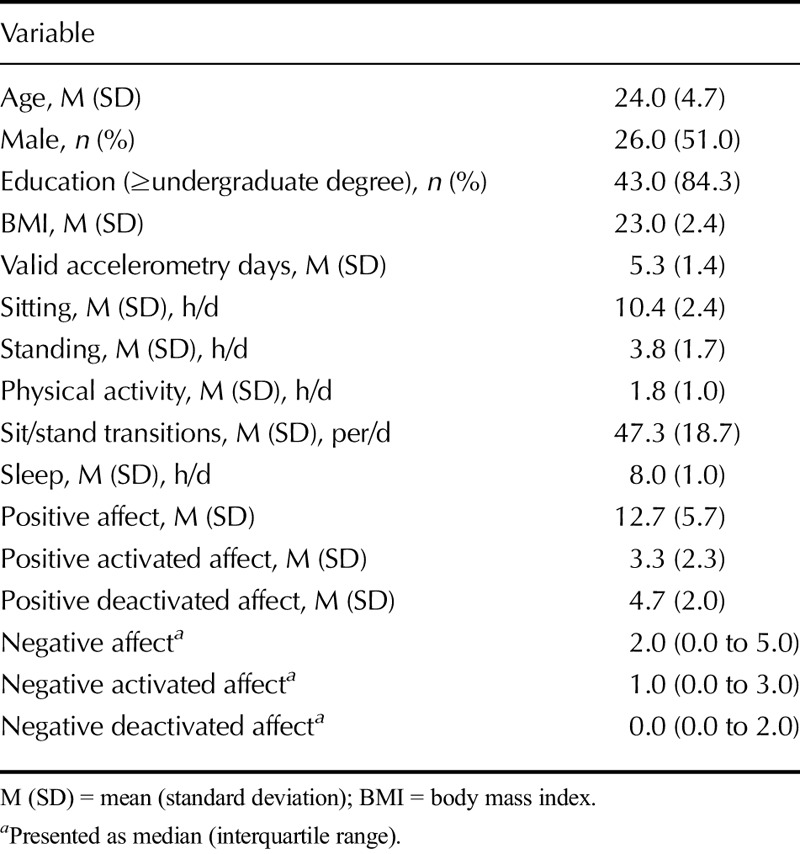
Sample Characteristics (*N* = 51)

### Associations With Positive Affect

In multilevel models, a borderline significant between-person association was found between higher levels of physical activity and higher positive affect after adjusting for age, sex, BMI sleep duration, and education (*B* = 1.85, 95% CI = −0.25 to 3.94). There were no between-person associations between sitting time, sit-to-stand transitions, and standing time with positive affect in any of the models (*B* values ranged from 0.29 to −0.21 in adjusted models). No within-person associations were observed between activity variables and positive affect (*B* values ranged from 0.00 to 0.28 in adjusted models) (Table 2).

**TABLE 2:**
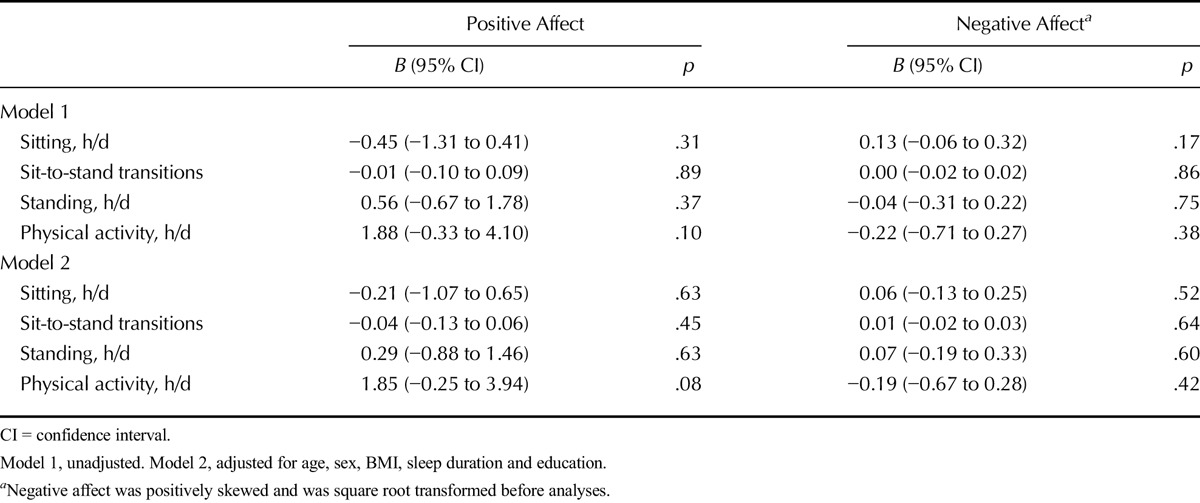
Between-Person Associations of Daily Sitting and Physical Activity (Averaged Over Week) With Positive and Negative Affect

### Associations With Negative Affect

No between-person associations were observed between activity variables and negative affect. However, significant within-person associations were found between physical activity and negative affect. A 1-hour increase in daily physical activity compared with average activity levels was associated with a decrease in negative affect over the same day in adjusted models (*B* = −0.11, 95% CI = −0.21 to −0.01) (Table 3).

**TABLE 3:**
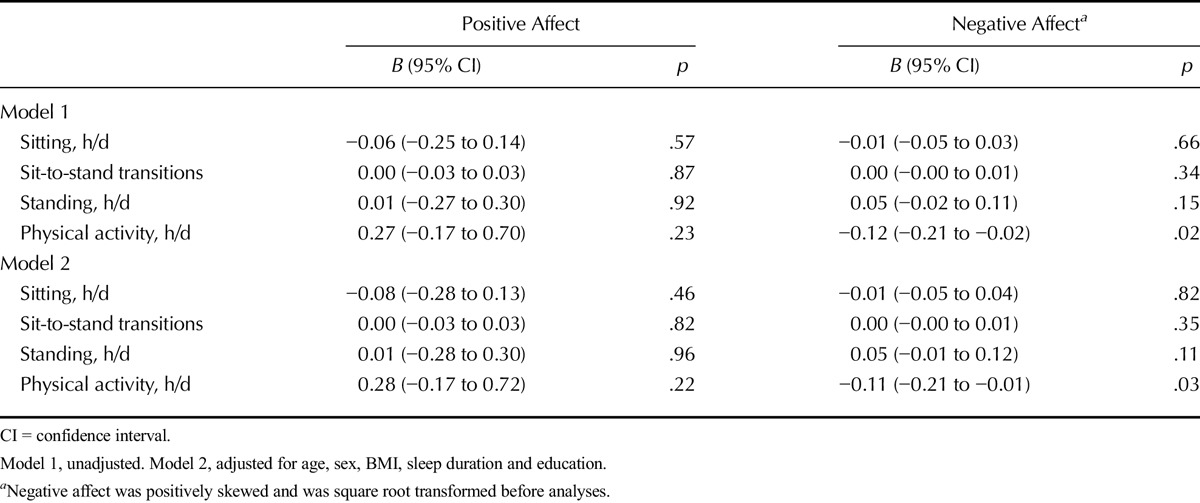
Within-Person Associations of Daily Sitting and Physical Activity (Centered on Subject-Specific Mean) With Positive and Negative Affect

In an additional analysis, we also explored associations of activity variables with activated and deactivated positive and negative affect. Vigor and calm subscales were used to describe activated and deactivated positive affect, respectively, whereas the anxiety and depression subscales described activated and deactivated negative affect. Between-person associations showed that physical activity was associated with deactivated positive affect (*B* = 0.71, 95% CI = 0.00 to 1.42), but not activated positive affect (Table S2, Supplemental Digital Content 1, http://links.lww.com/PSYMED/A399). Within-person associations showed that physical activity was associated with deactivated negative affect (*B* = −0.09, 95% CI = −0.18 to 0.00), but not activated negative affect (Table S3, Supplemental Digital Content 1, http://links.lww.com/PSYMED/A399). All associations remained significant after additionally adjusting for number of valid days and excluding participants with only 1 valid day (data not shown). Of note, these analyses revealed no significant between- or within-person associations between sleep duration and affective states (data not shown).

## DISCUSSION

The aim of this study was to determine the associations between objectively measured activity levels and daily assessments of affective states, distinguishing positive and negative daily affect, and between- and within-person relationships. The analyses revealed different between- (averaged daily activity across week) and within-person (individual-level daily fluctuations) associations between physical activity and affective states. Between-person associations showed only a borderline association between physical activity and greater positive affect and no associations with negative affect. Within-person associations showed that days with an additional hour of activity compared with average activity levels were associated with lower negative affect over the same day but were not associated with positive affect. This association remained significant after adjusting for sociodemographic characteristics and BMI. Overall, no within- or between-person associations were observed for time spent sitting, standing, and for number of sit-to-stand transitions with affective states.

These results are consistent with most previous studies using both self-reported and objectively measured physical activity showing that physical activity is associated with more favorable affective states (8,13,31–33). One previous study found that self-reported moderate-to-vigorous physical activity but only objectively measured light activity was associated with lower psychological distress (10). Although we were not able to differentiate between specific intensities of activity, we were able to capture time spent in different postural allocations objectively. In our study, only overall activity was favorably associated with affective states but not time spent standing or sit-to-stand transitions, which are reliably captured using the ActivPal. One of the main strengths of this study was the ability to determine the effects of acute physical activity fluctuations (within-person variation) on affective states. Previous studies have consistently reported an association between acute physical activity and increased positive affect (7). We did not find evidence for this association, but we did find that days with higher physical activity than average were associated with lower negative affect, which is consistent with some but not all previous studies (7). One possible explanation for this discrepancy may be that the dynamic changes to positive affect throughout the day were not captured in our study. A recent study using ecological momentary assessments in children showed that higher levels of moderate to vigorous physical activity across the day were associated with higher positive affect and lower negative affect 30 minutes later (13). Further affect measurements throughout the day may have captured the shorter-term effects of physical activity in our study. It may also be plausible that there would be stronger within-person associations for higher intensities of physical activity with positive affective states; however, we were unable to differentiate between intensities of physical activity in this study.

Previous studies have consistently reported adverse effects of sedentary time on mental well-being (10,34); however, in contrast, the present study found that both habitual and acute bouts of sitting time were not detrimental to affective states. As we measured sedentary time using appropriate objective measures, this may suggest that the physiological processes related to sitting are not an underlying cause of negative affect. Our sample was predominantly students who may not represent the types of sedentary behavior the rest of the population typically engage in. Plausibly, the types of sedentary behavior this sample engage in are not detrimental to affect. Some sedentary behaviors, such as listening to music, may act as a break from the stressors of daily life and therefore may improve affective state (35). This does not rule out the possibility that specific sedentary behaviors, such as TV viewing (36), may be detrimental to affect, but we do not have data to support this in the present study. Context may also drive associations between physical activity and affect. For example, greater positive affect is reported after acute bouts of physical activity with other people rather than acute bouts done alone. Similarly, lower negative affect is reported after acute bouts of outdoor activity compared with acute bouts of indoor activity (12). Importantly, the hip-mounted accelerometers used in the aforementioned studies and the majority of other studies in this field may be less precise for measuring sitting time than postural allocation accelerometers (17). Hip-mounted accelerometers are limited by their susceptibility for misclassifying sitting and standing (37). Using the ActivPal monitor, we could reliably quantify the associations of sitting, standing, and sit-to-stand transitions with affect.

We also explored associations of the activity variables with the activated and deactivated components of positive and negative affect. Previous studies have focused primarily on the associations with activated components of positive affect and have consistently shown that physical activity is associated with an increase in feelings of vigor and energy (38,39). However, our results only showed significant between-person associations between physical activity and deactivated positive affect, including feelings of calmness and being at ease. Some studies also suggest that physical activity is associated with specific components of negative affect (8,39). Similarly, we also found inverse associations between physical activity with deactivated components of negative affect but not activated components, which is consistent with previous studies showing that physical activity may reduce feelings of depression (2).

The major strength of this study is the novel use of postural allocation accelerometers combined with simultaneous repeated measurement of affective states. To our knowledge, this is the first study to examine the associations of sitting, standing, and sit-to-stand transitions with affective states. Although we found null associations in the present study, further research using these devices in more diverse populations is required to fully understand the associations between very light activities and affective states. The limitation of using these devices is that we were unable to distinguish between different intensities of physical activity, which may be differentially associated with affective states. Our study extends on the previous literature by examining how within-person fluctuations in activity levels are associated with affect. This revealed contrasting between- and within-person effects of physical activity with affect, suggesting that the acute and chronic effects of physical activity on mood may be operating via different mechanisms; increasing daily physical activity above usual levels may have acute benefits by lowering levels of negative affect, whereas habitual physical activity may be more important for positive affect. Importantly, lower within-person variability in affect compared with between-person may have impaired our ability to detect true within-person associations. Further limitations were that we were unable to determine the intensity of physical activity, and thus, we are unable to identify the optimal intensity for different affective states. Furthermore, taking only one affect measure throughout the day may have failed to capture the shorter-term effects of physical activity. Affective states measured at a single time point during the day may be biased toward reflecting the affective state at that moment. We are also unable to rule out the possibility of reverse causation. A number of studies using ecological momentary assessment have shown that affective states can also predict subsequent activity levels (40). Nevertheless, given that our mood assessments were completed in the evening, it is less likely that mood would have driven activity levels the following day. Reverse causation may also explain the borderline between-person association between physical activity and positive affect, as it may be possible that people with positive affective traits are subsequently more likely to be active. Alternatively, residual confounding from possible unmeasured factors related to affect and physical activity may explain these associations. Finally, our sample came from a predominantly healthy student population from University College London and had low caffeine consumption, so our results may not be generalizable to the wider population.

In conclusion, our study demonstrates that habitual and daily fluctuations in physical activity may be associated with improved affect. Increasing daily activity may be a potential strategy to acutely suppress negative affective states. Sitting, standing, and sit-to-stand transitions were not associated with affective states.

## Supplementary Material

SUPPLEMENTARY MATERIAL
