# Supplementary material for: Objectively Measured Daily Physical Activity and Postural Changes as Related to Positive and Negative Affect Using Ambulatory Monitoring Assessments
Source: Psychosom Med. 2017 Jun 20;79(7):792–7. doi: 10.1097/PSY.0000000000000485 (PMC5580380; doi:10.1097/PSY.0000000000000485)
Supplement: SUPPLEMENTARY MATERIAL [file psm-79-792-s001.docx]

**Table S1. Correlation matrix for mean activity variables**

|  | Sitting (hr/d) | Standing (hr/d) | Physical activity (hr/d) |
| --- | --- | --- | --- |
| Sitting (hr/d) | 1.00 |  |  |
| Standing (hr/d) | -0.67** | 1.00 |  |
| Physical activity (hr/d) | -0.70** | 0.40* | 1.00 |
| Sit-to-stand transitions | -0.21 | 0.09 | 0.34* |

*p<0.05;**p<0.001

**Table S2. Between-person associations of daily sitting and physical activity (averaged over week) with activated and deactivated positive affect**

|  | Activated Positive Affect | | Deactivated Positive Affect | |
| --- | --- | --- | --- | --- |
|  | B (95% CI) | p-value | B (95% CI) | p-value |
| Model 1 | | | | |
| Sitting (hr/d) | -0.07 (-0.39, 0.25) | 0.68 | -0.18 (-0.47, 0.09) | 0.20 |
| Sit-to-stand transitions | 0.00 (-0.04, 0.04) | 0.99 | 0.00 (-0.03, 0.03) | 0.96 |
| Standing (hr/d) | 0.29 (-0.16, 0.73) | 0.21 | 0.15 (-0.25, 0.56) | 0.46 |
| Physical activity (hr/d) | 0.41 (-0.42, 1.24) | 0.33 | 0.69 (-0.04, 1.42) | 0.07 |
| Model 2 | | | | |
| Sitting (hr/d)^a^ | -0.02 (-0.34, 0.30) | 0.90 | -0.13 (-0.43, 0.16) | 0.38 |
| Sit-to-stand transitions | -0.02 (-0.05, 0.02) | 0.35 | 0.00 (-0.03, 0.03) | 0.91 |
| Standing (hr/d) | 0.20 (-0.24, 0.63) | 0.38 | 0.07 (-0.33, 0.48) | 0.72 |
| Physical activity (hr/d)^b^ | 0.46 (-0.33, 1.26) | 0.25 | 0.71 (0.00, 1.42) | 0.05 |

Model 1, unadjusted. Model 2, controlled for age, sex, BMI, sleep duration and education.

**Table S3. Within-person associations of daily sitting and physical activity (averaged over week) with activated and deactivated negative affect**

|  | Activated Negative Affect ^a^ | | Deactivated Negative Affect ^a^ | |
| --- | --- | --- | --- | --- |
|  | B (95% CI) | p-value | B (95% CI) | p-value |
| Model 1 | | | | |
| Sitting (hr/d) | -0.01 (-0.04, 0.03) | 0.58 | 0.00 (-0.04, 0.04) | 0.90 |
| Sit-to-stand transitions | 0.00 (-0.00, 0.01) | 0.36 | 0.00 (-0.00, 0.01) | 0.61 |
| Standing (hr/d) | 0.02 (-0.03, 0.08) | 0.37 | 0.04 (-0.02, 0.09) | 0.19 |
| Physical activity (hr/d) | -0.06 (-0.14, 0.02) | 0.12 | -0.09 (-0.18, 0.00) | 0.04 |
| Model 2 | | | | |
| Sitting (hr/d) | 0.00 (-0.04, 0.03) | 0.83 | 0.00 (-0.04, 0.04) | 0.95 |
| Sit-to-stand transitions | 0.00 (-0.00, 0.01) | 0.35 | 0.00 (-0.00, 0.01) | 0.61 |
| Standing (hr/d) | 0.03 (-0.02, 0.08) | 0.29 | 0.04 (-0.16, 0.10) | 0.17 |
| Physical activity (hr/d) | -0.06 (-0.14, 0.02) | 0.13 | -0.09 (-0.18, 0.00) | 0.04 |

^a^ Negative affect was positively skewed and was square root transformed prior to analyses

Model 1, unadjusted. Model 2, controlled for age, sex, BMI, sleep duration and education.
